# Comparison of *Leishmania killicki* (syn. *L*. *tropica*) and *Leishmania tropica* Population Structure in Maghreb by Microsatellite Typing

**DOI:** 10.1371/journal.pntd.0004204

**Published:** 2015-12-08

**Authors:** Dhekra Chaara, Anne- Laure Bañuls, Najoua Haouas, Loïc Talignani, Patrick Lami, Habib Mezhoud, Zoubir Harrat, Jean-Pierre Dedet, Hamouda Babba, Francine Pratlong

**Affiliations:** 1 Laboratoire de Parasitologie-Mycologie Médicale et Moléculaire (code LR12ES08), Département de Biologie Clinique B, Faculté de Pharmacie, Université de Monastir, Tunisie; 2 Centre National de Référence des Leishmanioses, Département de Parasitologie-Mycologie, CHRU de Montpellier, Université de Montpellier, Montpellier, France; 3 UMR MIVEGEC (CNRS 5290-IRD 224-Université de Montpellier), Montpellier, France; 4 College of Applied Medical Sciences, Clinical Laboratory Sciences Department, University of Hail, Hail, Saudi Arabia; 5 Laboratoire d'éco-épidémiologie Parasitaire et Génétique des Populations. Institut Pasteur d'Algérie, Dély Ibrahim, Algeria; US Food and Drug Administration, UNITED STATES

## Abstract

*Leishmania* (*L*.) *killicki* (syn. *L*. *tropica*), which causes cutaneous leishmaniasis in Maghreb, was recently described in this region and identified as a subpopulation of *L*. *tropica*. The present genetic analysis was conducted to explore the spatio-temporal distribution of *L*. *killicki* (syn. *L*. *tropica*) and its transmission dynamics. To better understand the evolution of this parasite, its population structure was then compared with that of *L*. *tropica* populations from Morocco. In total 198 samples including 85 *L*. *killicki* (syn. *L*. *tropica*) (from Tunisia, Algeria and Libya) and 113 *L*. *tropica* specimens (all from Morocco) were tested. Theses samples were composed of 168 *Leishmania* strains isolated from human skin lesions, 27 DNA samples from human skin lesion biopsies, two DNA samples from *Ctenodactylus gundi* bone marrow and one DNA sample from a *Phlebotomus sergenti* female. The sample was analyzed by using MultiLocus Enzyme Electrophoresis (MLEE) and MultiLocus Microsatellite Typing (MLMT) approaches. Analysis of the MLMT data support the hypothesis that *L*. *killicki* (syn. *L*. *tropica*) belongs to the *L*. *tropica* complex, despite its strong genetic differentiation, and that it emerged from this taxon by a founder effect. Moreover, it revealed a strong structuring in *L*. *killicki* (syn. *L*. *tropica*) between Tunisia and Algeria and within the different Tunisian regions, suggesting low dispersion of *L*. *killicki* (syn. *L*. *tropica*) in space and time. Comparison of the *L*. *tropica* (exclusively from Morocco) and *L*. *killicki* (syn. *L*. *tropica*) population structures revealed distinct genetic organizations, reflecting different epidemiological cycles.

## Author Summary

*Leishmania killicki* (syn. *L*. *tropica*) was discovered in 1986. Few studies have been conducted on this parasite exclusively described in Maghreb. Consequently, many elements on its epidemiology, transmission, population structure and dynamics remain unknown.

To better understand the evolution of this parasite, its population structure has been compared with that of *L*. *tropica* populations from Morocco using Multilocus Enzyme Electrophoresis (MLEE) and MultiLocus Microsatellite Typing (MLMT) typing. MLMT data support the hypothesis that *L*. *killicki* (syn. *L*. *tropica*) belongs to the *L*. *tropica* complex despite the strong genetic differentiation between them. Despite the probable recent divergence between *L*. *killicki* (syn. *L*. *tropica*) and *L*. *tropica*, they seem to evolve differently. Indeed, *L*. *killicki* (syn. *L*. *tropica*) appears slightly polymorphic and highly structured in space and time, while *L*. *tropica* was genetically heterogeneous, slightly structured geographically and temporally. The different population structures revealed distinct genetic organizations, reflecting different epidemiological cycles. Several parameters could explain these opposite epidemiological and genetic patterns such as ecosystems, vectors and reservoirs.

## Introduction

Leishmaniases are vector-borne diseases caused by several *Leishmania* species that cycle between their phlebotomine sandfly vectors and mammalian reservoir hosts [1]. *Leishmania* parasites, like many other microorganisms, have a high adaptation capacity that allows them to invade and survive in various ecosystems. The spread of a parasitic genotype or group of genotypes in new ecosystems can lead to population differentiation. Consequently, new *Leishmania* taxa have regularly been described during the last decades [2–4]. *Leishmania killicki* could be considered as a typical example of this evolutionary process. Rioux et al. [5] identified this parasite in the Tataouine province (South Eastern Tunisia) for the first time in 1980. Then, sporadic cases were reported in Kairouan and Sidi Bouzid (Center of Tunisia), Gafsa (South Western Tunisia) and Séliana (Northern Tunisia) [6–8]. Besides Tunisia, this taxon was described in Libya [9] and Algeria [10–12]. The probable zoonotic transmission of this parasite, with the *Ctenodactylus gundi* rodent as reservoir and *Phlebotomus* (*P*.) *sergenti* as vector, was suggested but needs to be confirmed [13–17].

Data on *L*. *killicki* are scarce and the few available studies mainly focused on the detection and identification of this taxon using isoenzymatic or genetic approaches (PCR-RFLP, PCR-sequencing and PCR-SSCP) [18–21]. The isoenzymatic characterization using the MultiLocus Enzyme Electrophoresis (MLEE) technique identified four zymodemes for *L*. *killicki*. Zymodeme MON-8 (the most frequently identified) was found in isolates from Tunisia and Libya [5, 9]; zymodemes MON-301 and MON-306 were identified in Algeria [10, 11, 18], and MON-317 was characterized in Tunisia for the first time [22]. In a recent taxonomic study, we confirmed that *L*. *killicki* is included within the *L*. *tropica* complex and we suggested calling it *L*. *killicki* (syn. *L*. *tropica*) [22].

Nevertheless, *L*. *killicki* (syn. *L*. *tropica*) epidemiology, transmission dynamics and why it is essentially described in Tunisia are still not well understood. The specific objective of this study was to provide new insights on the molecular epidemiology and transmission of *L*. *killicki* (syn. *L*. *tropica*). To this aim, we carried out a genetic study based on the analysis of nine microsatellite loci by MultiLocus Microsatellite Typing (MLMT) in a sample of 198 isolates from different Maghreb regions to explore the population structure of *L*. *killicki* (syn. *L*. *tropica*) and to compare the data with those of *L*. *tropica* populations from Morocco.

## Materials and Methods

In the “Materials and Methods” and “Results” sections, *L*. *killicki* has been used at the place of *L*. *killicki* (syn. *L*. *tropica*) for easy reading.

### Geographic origin and isolation period of the *Leishmania* samples

A total of 198 samples were included in this study. They were composed by 154 *Leishmania* strains selected from the *Leishmania* collection of Montpellier, France (BRC-Leish, BioBank N° BB-0033-00052) and 44 samples collected by the research group of the Laboratoire de Parasitologie—Mycologie Médicale et Moléculaire (Monastir, Tunisia) during epidemiological investigations. These samples belonged to *L*. *killicki* (n = 85) and *L*. *tropica* (n = 113) and were identified over a period of 34 years (from 1980 to 2013). *L*. *killicki* samples were collected in Algeria (n = 7), Tunisia (n = 77) and Libya (n = 1). All the *L*. *tropica* strains were from Morocco since we have recently suggested that *L*. *killicki* and *L*. *tropica* from Morocco could have originated from a same *L*. *tropica* ancestor.

Among the 198 samples, 168 were isolates from infected patients (Morocco [n = 113]; Tunisia [n = 47]; Algeria [n = 7]; Libya [n = 1]), 27 were DNA samples from human skin lesion biopsies (Tunisia), two were DNA samples from *Ctenodactylus gundi* bone marrow (Tunisia) and one was a DNA sample from a *Phlebotomus sergenti* female (Tunisia) (see supplementary data S1 Table). The *L*. *killicki* samples from Tunisia (n = 77) and the *L*. *tropica* samples Morocco (n = 113) were classified according to the area and period of isolation (S1 Fig).

### Strain identification

Although some of the isolates included in this study were previously characterized [5, 7, 9, 10, 18, 20, 23, 24], they were all (n = 168) analyzed again at the Centre National de Référence des Leishmanioses (CNRL), Montpellier (France) using the MLEE technique and 15 enzymatic systems, according to Rioux et al. [25].

### DNA extraction and sample identification

Genomic DNA was extracted from the isolates using the QIAamp DNA Mini Kit, according to the manufacturer’s instructions, and eluted in 150 μl of AE buffer. The DNA samples from the 27 human skin biopsies, the two *C*. *gundi* and the *P*. *sergenti* were identified by polymerase chain reaction (PCR) amplification followed by digestion with BstU1 and Taq1, according to Haouas et al. [19]. The produced fragments were separated by electrophoresis on 3% agarose gels and compared with those of the WHO reference strains of *L*. *major* MON-25 (MHOM/MA/81/LEM265), *L*. *infantum* MON-1 (MHOM/FR/78/LEM75) and *L*. *killicki* MON-8 (MHOM/TN/80/LEM163).

### Microsatellite genotyping

First, few randomly selected *L*. *killicki* (n = 10) and *L*. *tropica* (n = 25) strains were genotyped by amplifying the 21 microsatellite loci already used by Schwenkenbecher et al. [26] in order to select the best markers. All 21 loci could be amplified in the *L*. *tropica* samples. Conversely, only nine loci (six described by Schwenkenbechet et al. [27] and three by Jamjoom et al. [28]) were amplified in the tested *L*. *killicki* strains. These nine loci were used for genotyping the 198 samples under study (see supplementary S2 Table).

All samples were amplified using the PCR conditions described by Schwenkenbecher et al. [27]: 2 min at 94°C and then 40 cycles of 94°C for 30 s, annealing temperature of each locus-specific primer set (2) for 30 s, 72°C for 1 min and a final extension step of 72°C for 10 min. The amplification products were visualized on 1.5% agarose gels. Multiplex genotyping was done using 1 μl of PCR-amplified DNA added to the Genescan 500LIZ internal size standard and 13.5μl of formamide in an automated sequencer. Genotyping data were analyzed with the Genemapper software v.4.0 to determine the fragment sizes.

### Data analysis

Fstat v. 2.9.3.2 [29], updated from Goudet [30], was used for statistical analysis of the sample genetic polymorphism based on Nei’s unbiased estimator of genetic diversity (*H*_*s*_) [31], the number of alleles per locus *(N*) and the mean allelic richness.

The same software was also used for calculating the Wright’s F statistics [32] according to the Weir and Cockerham’s method [33]. The *F*_st_ coefficient reflects the inbreeding that results from the subdivision of the population into sub-populations of limited size, and measures the genetic differentiation between sub-populations. It varies between 0 and 1; values > 0.25 reflect a high genetic differentiation [34]. *F*_st_ is considered significant when the *p-*value is ≤ 0.05. The *F*_*is*_ coefficient estimates the inbreeding of individuals due to the local non-random union of gametes in each subpopulation. *F*_*is*_ values range between -1 and 1. A negative value indicates an excess of heterozygotes, a positive value corresponds to heterozygote deficiency. Genotypes obtained from the concatenated sequences of the nine microsatellite loci were used to calculate the global genotypic diversity *D*_g_ (*D*_g_ = number of genotypes per population/total number of genotypes).

The Neighbor-Joining (NJ) phenetic tree was constructed using the MEGA 5.10 software [35] from a Cavalli-Sforza and Edwards [36] genetic distance matrix obtained using the POPULATIONS software (http://www.legs.cnrs-gif.fr/bioinfo/populations).

### Ethics statement

*Leishmania* strains were obtained from the *Leishmania* collection (BRC-Leish, Montpellier, France, BioBank N° BB-0033-00052) which is part of the French network of Biological Resources Centres for Microorganisms (FBRCMi). This parasite collection is isolated over a period of many years and is completely independent of patients from which strains were isolated. All samples taken from humans were anonymized.

## Results

### Isoenzymatic polymorphism of *Leishmania* strains

Isoenzymatic characterization of the *L*. *killicki* (n = 55) and *L*. *tropica* (n = 113) strains was performed to confirm (n = 166) or to identify (n = 2) their zymodemes. Ten zymodemes were obtained (three for *L*. *killicki* and seven for *L*. *tropica*). *L*. *killicki* was represented by three zymodemes. MON-8 was identified in 44 Tunisian isolates and in the Libyan sample, while MON-301 was found in the seven Algerian isolates (see S1 Table). The newly described zymodeme MON-317 was identified in three strains (MHOM/TN/2009/MET122, MHOM/TN/2010/MET300 and MHOM/TN/2010/MET301) isolated from the focus of Gafsa (South West of Tunisia) (S1 Table).

*L*. *tropica* was mainly represented by the zymodeme MON-102 (n = 76), followed by MON-113 (n = 22) and MON-107 (n = 6). The four remaining zymodemes were found only in few strains: MON-109 (n = 3), MON-112 (n = 3), MON-264 (n = 1) and MON-311 (n = 2) (S1 Table).

### Microsatellite analysis of the *Leishmania* strains

The 198 samples were amplified using primers for the nine investigated loci. Clear electropherograms and two alleles per locus and per sample were obtained (see supplementary data S3 Table). Twenty nine alleles were obtained, ranging from two for the GA1, GM2 and LIST7027 loci to five for the GA11 and LIST7036 loci (mean: 3.22 alleles per locus). The global genetic diversity was moderate (*H*_*s*_ = 0.261) and the global genotypic diversity was high (*D*_*g*_ = 0.53). The *F*_is_ values were positive at all loci and ranged from 0.120 for the LIST7040 locus to 0.920 for the GA6 locus (mean value = 0.664) (S2 Table).

### *L*. *killicki* population structure

Analysis of the genotyping data concerning all the *L*. *killicki* samples (n = 85) revealed 22 alleles ranging from a single allele for the GA1 and LIST7027 loci to five for the GA11 locus. The mean number of alleles per locus was 2.55 and the value of the mean allelic richness was 1.23. The global genetic diversity was low (*H*_*s*_ = 0.185) and the global genotypic diversity was moderate (*D*_*g*_ = 0.38) (Table 1).

**Table 1:** Allelic, genetic and genotypic variations of *L*. *killicki* and *L*. *tropica*

Taxon	Total number of alleles	Number of specific alleles	Mean number of alleles per locus	Mean allelic richness	*Hs*	*Dg* (Number of genotypes/ Total number of genotypes)
*L*. *killicki* (n = 85)	22	3	2.55	1.23	0.185	0.38 (41/106)
*L*. *tropica* (n = 113)	26	7	2.88	1.98	0.38	0.63 (66/106)

*Hs*, Nei's unbiased genetic diversity within samples; *Dg*, genotypic frequency

Comparison of the data for the *L*. *killicki* samples from Tunisia (n = 77) and from Algeria (n = 7) indicated that their genetic diversity was low (*H*_*s*_ = 0.215 for the Tunisian strains and *H*_*s*_ = 0.15 for the Algerian strains) and that the genetic differentiation between these populations was low, but significant (*F*_st_ = 0.11, *p* = 0.03) (Table 2). The *H*_*s*_ and *F*_st_ values were not calculated for *L*. *killicki* from Libya because only one specimen was available. Moreover, estimation of the genetic differentiation between the different Tunisian populations (strains from Gafsa, Tataouine and Kairouan-Séliana) and the Algerian samples showed that the genetic differentiation was important between the populations from Tataouine and Algeria (*F*_st_ = 0.34, *p* = 0.005) and lower but still significant between the samples from Gafsa and Algeria (*F*_st_ = 0.09, *p* = 0.01). No genetic differentiation was found between the Kairouan-Séliana and Algerian populations (*F*_st_ = 0.1, *p* = 0.18), possibly due to the small number of specimens from Kairouan-Séliana (n = 3) (Table 2).

**Table 2:** Genetic differentiation by locality and period of isolation between the *L*. *killicki* and *L*. *tropica* populations

Taxon	Populations	*F*_*st*_	*p*
*L*. *killicki*	Tunisia (n = 77)—Algeria (n = 7)	0.11	0.033
	Algeria (n = 7)—Gafsa (n = 37)	0.09	0.01
	Algeria (n = 7) -Tataouine (n = 37)	0.34	0.005
	Algeria (n = 7)—Kairouan Séliana (n = 3)	0.1	0.18
	Tataouine (n = 37) -Gafsa (n = 37)	0.3	0.002
	Tataouine (n = 37) -Kairouan Séliana (n = 3)	0.14	0.08
	Gafsa (n = 37) -Kairouan Séliana (n = 3)	≈ 0	1
	[1980, 1989] (n = 27)—[2000, 2009] (n = 24)	0.3	0.008
	[1980, 1989] (n = 27)—[2009, 2013] (n = 25)	0.35	0.008
	[2000, 2009] (n = 24)—[2010, 2013] (n = 25)	0.0018	0.2
	Tataouine [1980, 1989] (n = 27)—Gafsa [2000, 2009] (n = 13)	0.35	0.002
	Tataouine [1980, 1989] (n = 27)—Gafsa [2010, 2013] (n = 24)	0.44	0.002
	Tataouine[2000, 2009] (n = 9)—Gafsa[2010, 2013] (n = 24)	0.14	0.04
	Gafsa [2000, 2009] (n = 13)–Tataouine [2000, 2009] (n = 9)	0.02	0.1
	Gafsa [2000, 2009] (n = 13)–Gafsa [2010, 2013] (n = 24)	0.06	0.12
	Tataouine [1980, 1989] (n = 27)–Tataouine [2000, 2009] (n = 9)	0.23	0.002
*L*. *tropica*	Azilal (n = 52)—Essaouira (n = 40)	0.05	0.05
	Azilal (n = 52)—Ouarzazate (n = 8)	0.03	0.16
	Azilal (n = 52)—Salé (n = 3)	0.01	0.43
	Azilal (n = 52)—Taza (n = 10)	≈ 0	0.68
	Essaouira (n = 40)—Ouarzazate (n = 8)	0.025	0.035
	Essaouira (n = 40)—Salé (n = 3)	0.024	0.24
	Essaouira (n = 40)–Taza (n = 10)	0.04	0.045
	Ouarzazate (n = 8)—Salé (n = 3)	0.04	0.31
	Ouarzazate (n = 8)—Taza (n = 10)	0.02	0.43
	Salé (n = 3)—Taza (n = 10)	≈ 0	0.5
	[1980, 1989] (n = 44)—[1990, 1999] (n = 36)	0.03	0.045
	[1980, 1989] (n = 44)—[2000, 2009] (n = 33)	0.17	0.005
	[1990, 1999] (n = 36)—[2000, 2009] (n = 33)	0.13	0.005

*Fst*, coefficient of the genetic differentiation; *p*, probability

Analysis of the genetic diversity within the different *L*. *killicki* populations from Tunisia showed that the Gafsa strains (*H*_*s*_ = 0.22) were more polymorphic than the Tataouine strains (*H*_*s*_ = 0.15). The *H*_*s*_ value for the Kairouan-Séliana population was certainly biased because of the low number of strains and was not considered in this analysis. Finally, the genetic differentiation between the Gafsa and Tataouine populations was also high (*F*_st_ = 0.3, *p* = 0.002) (Table 2).

Analysis of the genetic diversity of the *L*. *killicki* samples classified based on the time of isolation indicated that specimens isolated during the 1980–1989 period were less diversified (*H*_*s*_ = 0.13) than those isolated between 2000 and 2009 (*H*_*s*_ = 0.24) or 2010 and 2013 (*H*_*s*_ = 0.2). The *H*_*s*_ value was not calculated for the 1990–1999 period because only one strain was collected during that time window. Genetic differentiation was important between the population isolated during the 1980–1989 period and the other populations. Conversely, no genetic differentiation was found between the populations collected between 2000 and 2009 and between 2010 and 2013 (Table 2). The *F*_st_ value was not estimated for the 1990–1999 window because only one strain was isolated in that period.

Analysis of the genetic diversity of the *L*. *killicki* samples classified based on the region and time of isolation revealed relatively higher *H*_*s*_ values for the specimens collected in Gafsa at different times (*H*_*s*_ Gafsa [2000–2009] = 0.26, *H*_*s*_ Gafsa [2010–2013] = 0.28) than for those collected in Tataouine (*H*_*s*_ Tataouine [1980–1989] = 0.13, *H*_*s*_ Tataouine [2000–2009] = 0.16). The Kairouan-Séliana strains isolated at different periods and the Tataouine strains collected during the 1990–1999 period were not included in this analysis due to their limited number.

Analysis of the genetic differentiation between these populations showed high *F*_st_ values that reflected temporal and geographical differences. However, a moderate genetic differentiation was found in the samples collected in Tataouine between 1980 and 1989 and between 2000 and 2009 and no genetic differentiation was observed between the strains isolated in Gafsa and Tataouine during the 2000–2009 period (Table 2).

**Fig 1:**
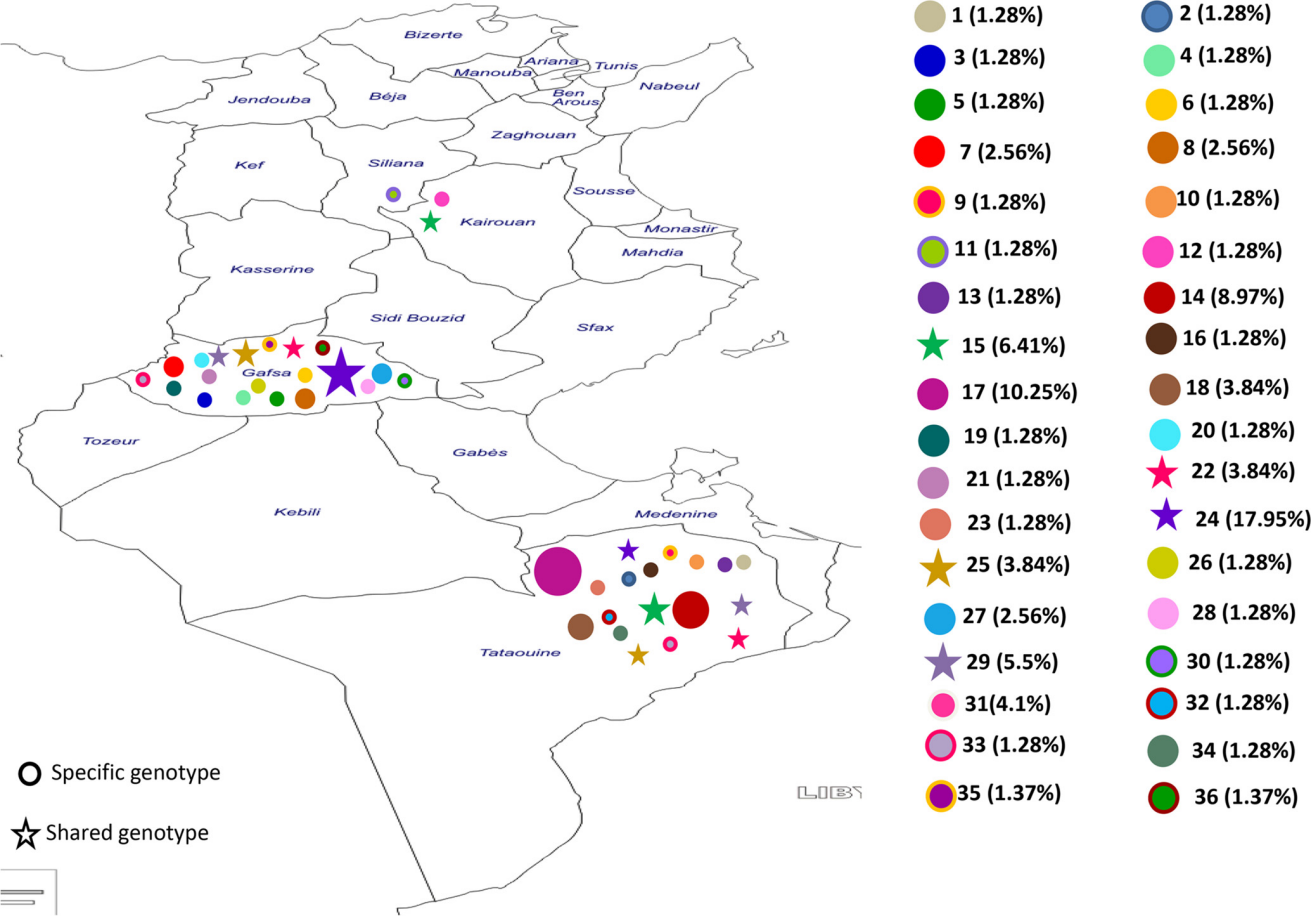
Distribution of the 36 *L*. *killicki* genotypes in Tunisia.

**Fig 2:**
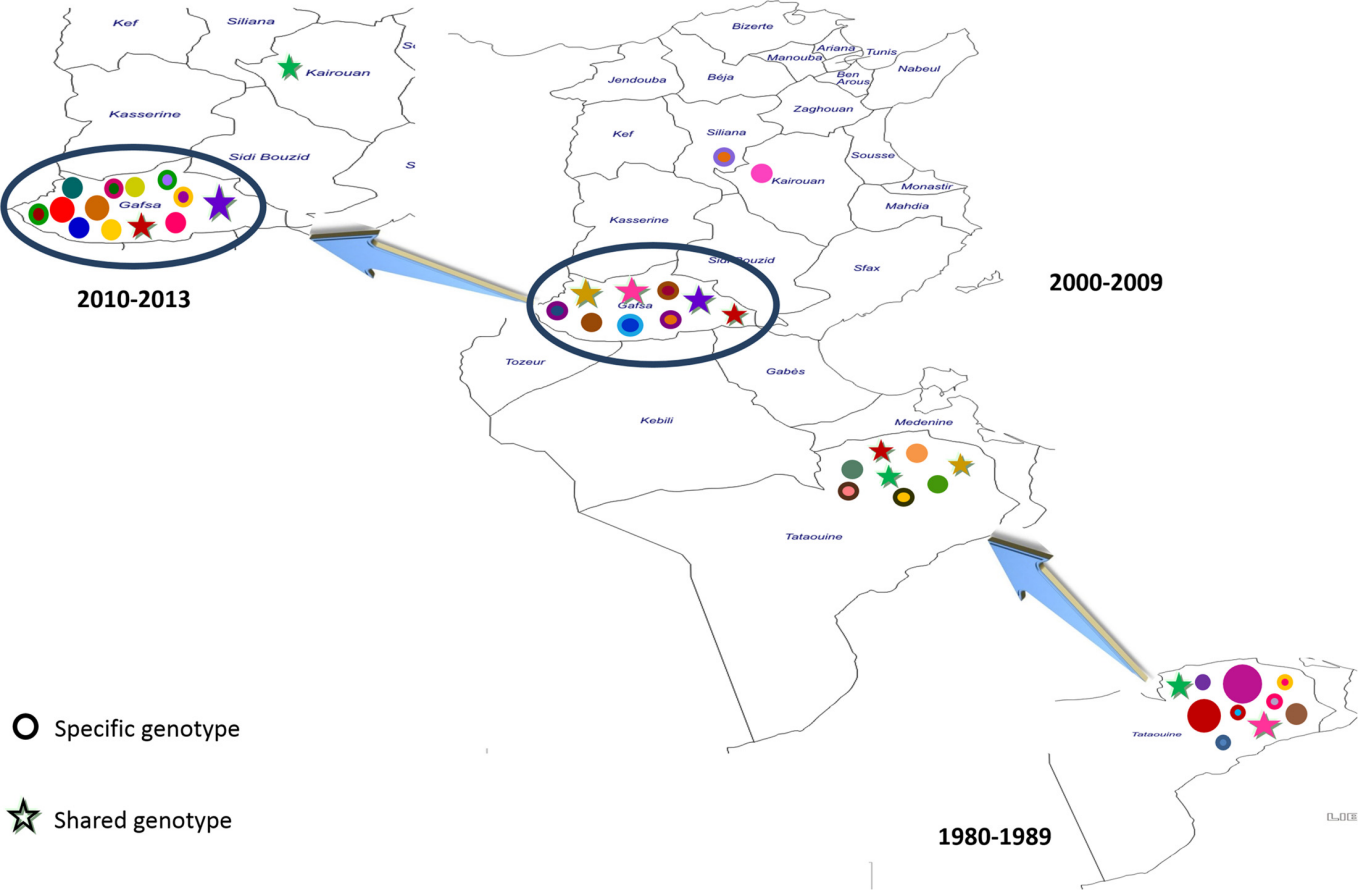
Spatio-temporal evolution of *L*. *killicki* genotypes in Tunisia (the key is the same as Fig 1).

Finally, thirty-six genotypes were found. Genotype 24 was the most frequent (17.95%) in the samples from Gafsa and Tataouine (Fig 1). Analysis of the genotype distribution in each focus and according to the time of isolation showed that most genotypes were specific to a locality or to a period of isolation (see Figs 1 and 2).

### *L*. *tropica* population structure

Twenty six alleles were identified ranging from two for the GA1 and LIST7027 loci and five for LIST7036. The mean number of alleles per locus was 2.88 and the mean allelic richness was 1.98. The global genetic diversity (*H*_*s*_ = 0.38) and genotypic diversity (*D*_*g*_ = 0.63) were high (Table 1).

Genetic diversity was also high when strains were classified according to the area of isolation in Morocco (*H*_*s*_ Azilal = 0.34, *H*_*s*_ Essaouira = 0.44, *H*_*s*_ Ouarzaezate = 0.44, *H*_*s*_ Taza = 0.38). For the strains from the locality of Salé, the *H*_*s*_ was not estimated because of their limited number (n = 3). Genetic differentiation was mainly not observed between strains from different localities; however, few *F*_st_ values were significantly different, although they were very low (from *F*_st_ = 0.025, *p* = 0.035 to *F*_st_ = 0.05, *p* = 0.05) (Table 2).

Genetic diversity was high also when the *L*. *tropica* strains were classified according to the period of isolation ([1980–1989] *H*_*s*_ = 0.35; [1990–1999] *H*_*s*_ = 0.35; [2000–2009] *H*_*s*_ = 0.43), whereas genetic differentiation was moderate but significant (Table 2).

### Comparison of the population structures of *L*. *killicki* and *L*. *tropica*

**Fig 3:**
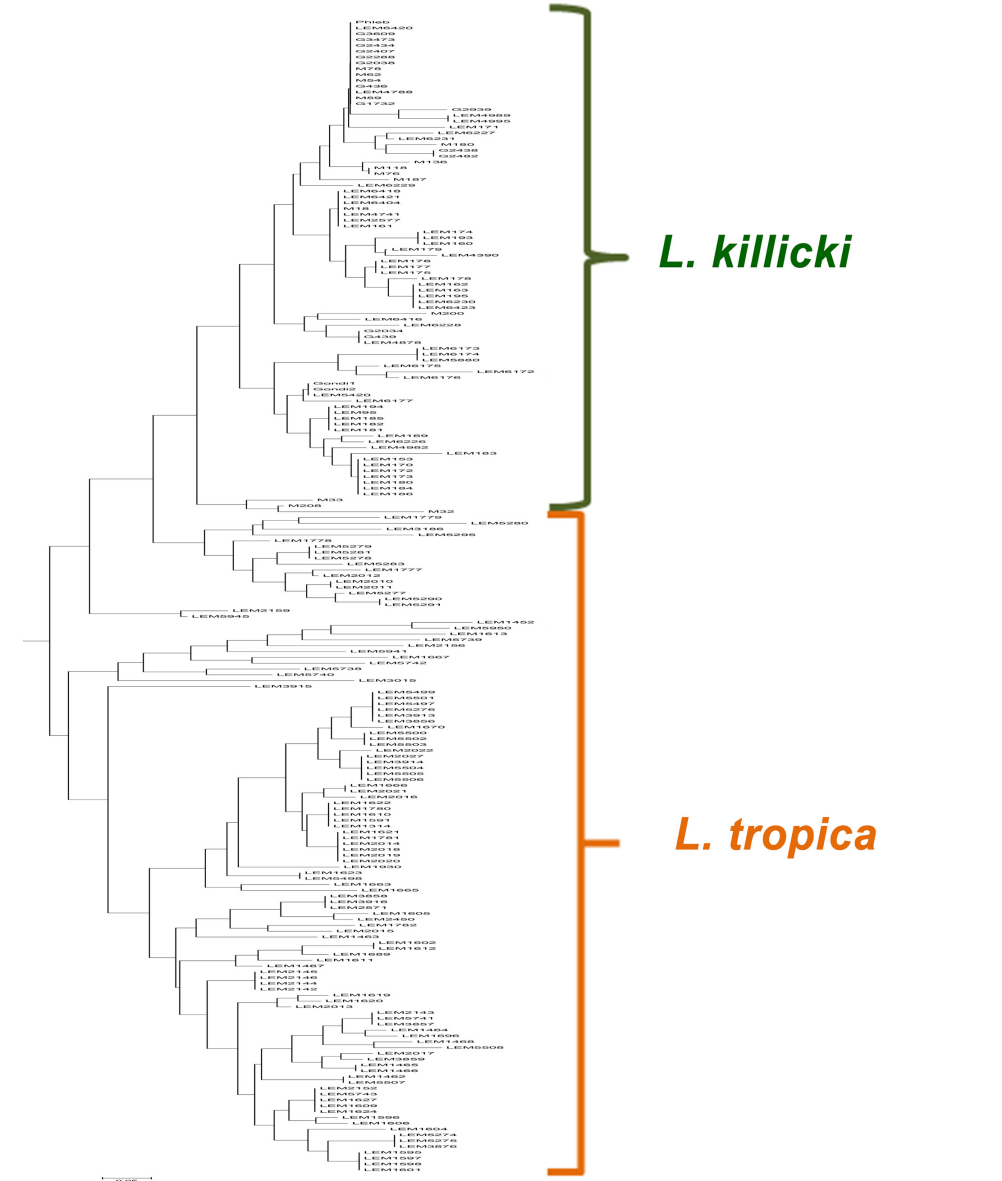
Neighbor-joining tree of the 198 samples based on the genetic distance relationships among the nine microsatellites tested in this study.

Comparison of the genotyping data showed strong genetic links between the *L*. *killicki* and *L*. *tropica* populations with 19 shared alleles among the 29 detected. Moreover, the NJ tree showed that *L*. *killicki* forms a monophyletic cluster inside the *L*. *tropica* complex (see Fig 3).

Overall, the *L*. *killicki* population was characterized by lower genetic and genotypic diversity, fewer alleles per locus and lower allelic richness than the *L*. *tropica* population (Table 1). Analysis of the population structure showed an important genetic differentiation between the *L*. *tropica* population and the entire *L*. *killicki* sample (*F*_*st*_ = 0.53, *p* = 0.01) and also between the *L*. *tropica* population and the *L*. *killicki* populations from Tunisia [*F*_st_ = 0.53, *p* = 0.01] and from Algeria [*F*_st_ = 0.5, *p* = 0.01]) (Table 3). This result was confirmed also when the *L*. *tropica* population was compared with the *L*. *killicki* populations from the different locations in Tunisia (Gafsa, Tataouine, Kairouan Séliana) (*F*_*st*_ > 0.4, *p* < 0.05) (Table 3).

**Table 3:** Genetic differentiation between *L*. *killicki* and *L*. *tropica* populations from Maghreb

Populations	*F*_*st*_	*p*
Morocco (*L*. *tropica*) (n = 113) -Tunisia (*L*. *killicki)* (n = 77)	0.53	0.01
Morocco (*L*. *tropica*) (n = 113)—Algeria (*L*. *killicki*) (n = 7)	0.5	0.01
Morocco (*L*. *tropica*) (n = 113)—Gafsa (*L*. *killicki*) (n = 37)	0.5	0.005
Morocco (*L*. *tropica*) (n = 113) -Tataouine (*L*. *killicki*) (n = 37)	0.55	0.005
Morocco (*L*. *tropica*) (n = 113) -Kairouan Séliana (*L*. *killicki*) (n = 3)	0.47	0.005

*Fst*, coefficient of the genetic differentiation; *p*, probability

## Discussion

Despite a great knowledge on *Leishmania* parasites, many taxa, such as *L*. *killicki* (syn. *L*. *tropica*), are still not completely characterized. The main objective of this study was to understand the epidemiology and transmission dynamics of *L*. *killicki* (syn. *L*. *tropica*) by analyzing its population structure and by comparing the genetic patterns of *L*. *killicki* (syn. *L*. *tropica*) and *L*. *tropica* populations in Maghreb.

The comparison of *L*. *killicki* (syn. *L*. *tropica*) and *L*. *tropica* revealed a strong genetic differentiation associated with a lower genetic polymorphism within *L*. *killicki* (syn. *L*. *tropica*). Furthermore, the NJ tree showed that *L*. *killicki* (syn. *L*. *tropica*) creates a homogeneous and monophyletic cluster within the *L*. *tropica* complex. These data support the recently obtained MultiLocus Sequence Typing (MLST) results [22] suggesting that *L*. *killicki* (syn. *L*. *tropica*) emerged from *L*. *tropica* by a founder effect. The strong genetic differentiation indicates an independent evolution and an absence of gene flow between the two taxa after the founder event. The geographic distance and the ecological barriers between Morocco (area of isolation of all *L*. *tropica* specimens) and Tunisia, Libya and Algeria (regions of origin of all *L*. *killicki* (syn. *L*. *tropica*) samples) as well as the different transmission cycles can explain this diversification. Maghreb countries are essentially separated by mountains and the Sahara desert that could prevent the circulation and migration of *Leishmania* vectors and reservoirs. Furthermore, *L*. *killicki* (syn. *L*. *tropica*) transmission cycle is most probably zoonotic [14, 15], whereas that of *L*. *tropica* appears to be both zoonotic or anthroponotic [24].

The comparison of *L*. *killicki* (syn. *L*. *tropica*) samples from Tunisia and Algeria revealed also a differentiation within this taxon, but lower than the one detected with *L*. *tropica*. This result supports the idea that *L*. *killicki* (syn. *L*. *tropica*) spread recently and may be still spreading between the different countries after the founder event. It is not known yet where the *L*. *tropica* subpopulation emerged to generate *L*. *killicki* (syn. *L*. *tropica*), but the number of reported cases suggests Tunisia. Despite the low sample size from Algeria, we detected a strong and significant genetic differentiation between the population from Tataouine and the samples from Algeria and a low genetic differentiation between the Gafsa and Algerian populations. These results seem to indicate a more recent diversification between the Gafsa and Algerian populations, supporting the hypothesis of a recent *L*. *killicki* (syn *L*. *tropica*) dispersion from Gafsa to Algeria. Conversely, the only isolate from Libya is genetically closer to the Tataouine than to the Gafsa population. This pattern is in agreement with the geographical distances/characteristics of these regions. Indeed, the mountains in the Gafsa area, where the probable reservoir(s) of *L*. *killicki* (syn *L*. *tropica*) live(s), belong to the Atlas Mountain chains, while mountains in the Tataouine region are connected to the Libyan mountains.

Concerning the *L*. *killicki* (syn. *L*. *tropica*) populations from Gafsa and Tataouine, despite their low genetic diversity indices, they show a strong and significant genetic differentiation with a lower genetic diversity among the Tataouine samples. These data suggest that the Tataouine population is more recent and that these two populations are genetically isolated. The presence of geographical barriers separating the South West and South East of Tunisia (the Sahara desert and the Chott Djerid salt lake) could explain this structuring.

Analysis of the spatio-temporal evolution of *L*. *killicki* (syn. *L*. *tropica*) in Tunisia shows a low circulation of genotypes between the different populations not only in space, but also in time within a region. Based on this observation and because most isolates were from infected humans, we can hypothesize that *L*. *killicki* (syn. *L*. *tropica*) mainly circulates in the reservoir host *C*. *gundi* and humans are accidentally infected. This is in agreement with the zoonotic character of *L*. *killicki* (syn. *L*. *tropica*) compared to *L*. *tropica*, which is known to be an anthropozoonotic or zoonotic pathogen, according to the infection focus [24].

Comparison of *L*. *tropica* from Morocco and *L*. *killicki* (syn. *L*. *tropica*) from Tunisia revealed that the population structures of these two taxa are different. Indeed, *L*. *killicki* (syn. *L*. *tropica*) populations from Tunisia showed an important genetic differentiation and differences in terms of genetic diversity, whereas the *L*. *tropica* populations from Morocco were genetically more homogeneous and only slightly differentiated. These data suggest that *L*. *killicki* (syn. *L*. *tropica*) poorly disperses (except for rare migration events from a region to another) compared to *L*. *tropica* from Morocco. This finding might reflect different ecological patterns, such as epidemiological cycles, infection of the reservoirs or vector behavior.

To conclude, this detailed study on *L*. *killicki* (syn. *L*. *tropica*) population genetics allowed exploring the evolutionary history of this parasite and highlighting its different genetic patterns compared to *L*. *tropica*. Despite the probable recent divergence between these taxa, they seem to evolve differently in terms of epidemiological cycle and thus transmission dynamics. Particularly, this study supports the hypothesis of a zoonotic transmission cycle for *L*. *killicki* (syn. *L*. *tropica*). Our data also suggest that Gafsa could be the historical focus of *L*. *killicki* (syn. *L*. *tropica*), although the sample size from the other regions was too small to firmly validate this hypothesis.

It is now essential to study the *P*. *sergenti* vector populations in Tunisia and their susceptibility to *L*. *killicki* (syn. *L*. *tropica*) and the parasite biology in *C*. *gundi* to better understand the transmission cycle of this parasite.

Although for the moment, *L*. *killicki* (syn. *L*. *tropica*) should be still considered a *L*. *tropica* subpopulation, our analyses indicate that in the future, this taxon position may have to be reconsidered.

## Supporting Information

S1 Fig Maps of the Tunisian (a) and Moroccan (b) localities where *L*. *killicki* (syn. *L*. *tropica*) (Tunisia) and *L*. *tropica* (Morocco) samples were isolated and isolation periods. (TIF)

S1 Table Characteristics of the 198 *Leishmania* samples analyzed in this study. (XLS)

S2 Table Characteristics and genetic diversity of the nine microsatellite loci in the 198 samples of *L*. *killicki* and *L*. *tropica*. (XLS)

S3 Table The genotypes of each sample at each locus. (XLS)
