# Supplementary figures and images for: Comparison of Leishmania killicki (syn. L. tropica) and Leishmania tropica Population Structure in Maghreb by Microsatellite Typing
Source: PLoS Negl Trop Dis. 2015 Dec 8;9(12):e0004204. doi: 10.1371/journal.pntd.0004204 (PMC4672892; doi:10.1371/journal.pntd.0004204)

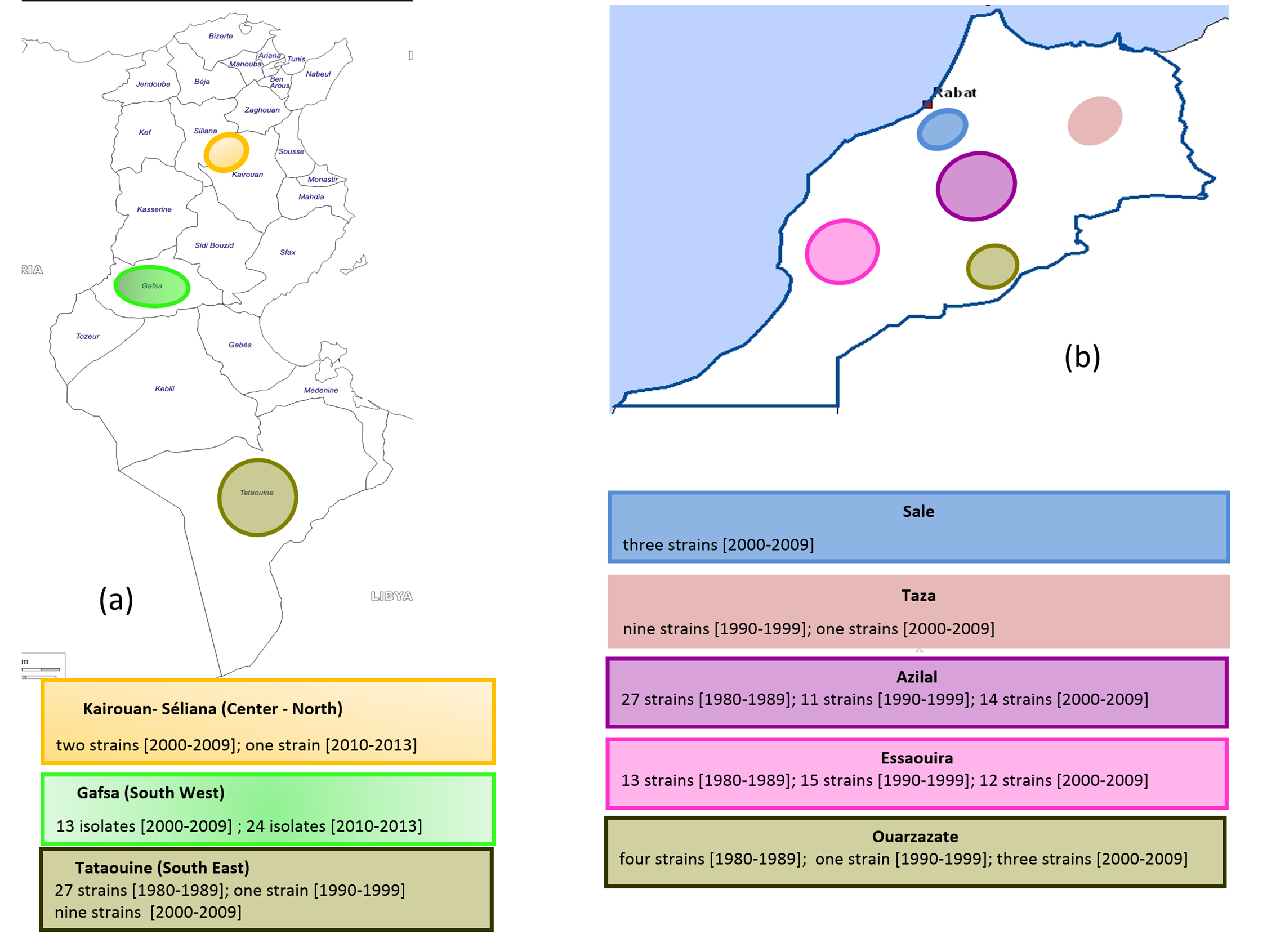

Supplement: S1 Fig — (TIF) [file pntd.0004204.s001.tif]
